# Low-dose aspirin for primary prevention of adverse pregnancy outcomes in twin pregnancies: an observational cohort study based on propensity score matching

**DOI:** 10.1186/s12884-021-04217-2

**Published:** 2021-11-22

**Authors:** Ying Ye, Li Wen, Xiyao Liu, Lan Wang, Yamin Liu, Richard Saffery, Mark D. Kilby, Chao Tong, Hongbo Qi, Philip Baker

**Affiliations:** 1 Department of Obstetrics, The First Affiliated Hospital of Chongqing Medical University, Chongqing, 400016 China; 2 State Key Laboratory of Maternal and Fetal Medicine of Chongqing Municipality, The First Affiliated Hospital of Chongqing Medical University, Chongqing, 400016 China; 3 Department of Obstetrics, Chongqing Women and Children’s Health Center, Chongqing, 401147 China; 4 Cancer, Disease and Developmental Epigenetics, Murdoch Children’s Research Institute, Parkville, Victoria 3052 Australia; 5 Department of Paediatrics, University of Melbourne, Parkville, Victoria 3052 Australia; 6 Institute of Metabolism and System Research, University of Birmingham, Edgbaston, UK; 7 Fetal Medicine Centre, Birmingham Women’s & Children’s Foundation Trust, Birmingham, B15 2TG UK; 8 College of Medicine, Biological Sciences and Psychology, University of Leicester, Leicester, LE1 7RH UK

**Keywords:** Low-dose aspirin, Twin pregnancy, Preeclampsia, Preterm birth, Fetal growth restriction, Postpartum hemorrhage

## Abstract

**Background:**

Since the effectiveness of low-dose aspirin (LDA) in twin pregnancies is uncertain, we aimed to preliminarily assess whether LDA is beneficial in preventing preeclampsia in twin pregnancies.

**Methods:**

This study is an observational study in two hospitals in China. Among 932 women, 277 in the First Affiliated Hospital of Chongqing Medical University were routinely treated with aspirin (100 mg daily) from 12 to 16 weeks to 35 weeks of gestational age, while 655 in Chongqing Health Center for Women and Children were not taking aspirin during pregnancy. We followed each subject and the individual details were recorded.

**Results:**

LDA significantly reduced the risk of preeclampsia (RR 0.48; 95% CI 0.24–0.95) and preterm birth 34 weeks (RR 0.50; 95% CI 0.29–0.86) and showed possible benefits to lower the rate of SGA babies (RR 0.74; 95% CI 0.55–1.00). Moreover, the risk of postpartum hemorrhage was not increased by LDA (RR 0.89; 95% CI 0.35–2.26).

**Conclusions:**

Treatment with low-dose aspirin in twin pregnancies could offer some protection against adverse pregnancy outcomes in the absence of significantly increased risk of postpartum hemorrhage.

**Trial registration:**

Chinese Clinical Trial Registry (ChiCTR); ChiCTR-OOC-16008203, Retrospectively registered date: April 1st, 2016;

**Supplementary Information:**

The online version contains supplementary material available at 10.1186/s12884-021-04217-2.

## Background

The prevalence of twin pregnancy is increasing, primarily as a result of the use of Assisted Reproductive Technologies (ART). Relative to singletons, twin pregnancies are associated with increased maternal and perinatal mortality and morbidity, and associated perinatal adverse outcomes, including preeclampsia (PE), small for gestational age (SGA) babies, and preterm birth (PTB) [1]. Recently, it has been reiterated that the incidence of PE in twin pregnancies is 3–4 times higher compared to singletons [2]. A clinical cohort study containing 321 twin pregnancies demonstrated the incidence of at least one twin with birthweight <10th centile was 47%, while at least one with birthweight <5th centile was 27% [3]. It is also reported that the percentage of PTB in twin pregnancies is significantly higher than in singletons, with 50–60% giving birth prior to 37 weeks [4]. The increasing morbidity and mortality associated with adverse pregnancy outcomes of PTB, PE, and SGA remain major concerns internationally. Although the etiology of these complications has not yet been fully elucidated, their development is generally associated with increased inflammation and/or hypoxia [5–8].

The well-described anti-inflammatory and anti-coagulation properties of aspirin have made it an appealing target for preventing gestational hypertensive disorders, but the specific mechanism is still unclear [9]. According to recent studies, we have a better understanding of the effects of aspirin on the prophylaxis of PE, PTB, and SGA [10–12]. Based on current data, the American College of Obstetricians and Gynecologists (ACOG) recommends daily low-dose aspirin (81 mg/day) in pregnancy to prevent adverse outcomes and while not increasing the risk of postpartum hemorrhage [13]. Another meta-analysis, however, concluded that a dose of aspirin between 100 and 150 mg was more beneficial, especially if initiated before 16 weeks [14]. The optimal dose has not yet reached a consensus. Unlike in singletons, few studies have explored the potential beneficial (or otherwise) effects of aspirin in twin pregnancies. The ACOG-guideline recommended pregnant women to take aspirin based on circumstantial evidence – according to the incidence of PE being much higher in twin pregnancies [13]. But there is a relative lack of direct evidence whether aspirin could reduce these adverse outcomes in twin pregnancies.

Given the limited data on twin pregnancies and conflicting results in singleton pregnancies, we undertook an observational cohort study based on real-world data. We aimed to explore the potential benefits of aspirin on twin pregnancies and provides a theoretical basis for further studies.

## Methods

This study was designed as an observational cohort based on real-world data in two centers from March 2016 to December 2018. We aimed to explore whether LDA can reduce the incidence of PE in twin pregnancies. Pregnant women with twins who ‘booked’ in the First Affiliated Hospital of Chongqing Medical University were routinely offered prophylaxis with low-dose aspirin (LDA group) before 16 weeks of gestational age and those recruited from Chongqing Health Center for Women and Children were not required to take aspirin and regarded as controls (NC group). This difference was based upon clinician preference in the two institutions.

Inclusion criteria were: Twin pregnancies diagnosed by ultrasound in the first visit (gestational age less than 16 weeks); 18–55 years old. Those who did not start their first antenatal care in the two centers but did give birth in these two hospitals were excluded in the final analysis due to incomplete records of prior medications.

### Processes

All women’s clinical, demographic, neonatal assessments, and outcomes data during childbirth were recorded in the Case Report Form (CRF). All information was concurrently recorded in both Hospital Information Systems (HIS) and Cohort Management Systems. Women (both in the LDA and in the NC group) were asked to have visits every 2 weeks before 28 weeks of gestational age, and every week after 28 weeks. In the LDA group, participants were administered aspirin from the day of registration. Aspirin 100 mg was taken per night from recruitment until 35 weeks of gestational age. The drug usage of women would be obtained orally and be recorded in the cohort management system at each follow-up point, and those participants who took extra drugs on their initiative would be removed from the study. The given doses of aspirin will be recorded in each follow-up point, afterward, the residual doses in the next follow-up point will be checked, and according to the interval days between the two adjacent follow-up points, the drug compliance could be calculated. According to the medical records, each woman in the LDA group was confirmed to have over 90% drug compliance. Incidence of PE and other adverse pregnancy outcomes were compared between LDA and control group.

### Statement about aspirin

Due to lack of consensus in using LDA for twin pregnancies, the two centers in this study carried out different policies on aspirin use for twin pregnancies and thus made this observational study feasible. Given that the specification of aspirin tablets in China is either 25 mg or 100 mg, and ACOG recommendation in pregnancy is 81 mg daily, 100 mg/d was prescribed to avoid under dosage.

### Outcome measures

Primary outcome was the incidence of PE and secondary outcomes included gestational age at birth (allowing assessment of PTB) and birthweight (allowing SGA-assessment). Clinical safety of aspirin was evaluated by the occurrence of antenatal or postpartum hemorrhage. PE was defined by blood pressure ≥ 140/90 mmHg associated with proteinuria (> 300 mg/day) or blood pressure ≥ 160/110 mmHg alone) after 20 weeks of gestational age [13, 15]. Blood pressure was measured by a mercury sphygmomanometer and urine samples were collected and tested by the clinical laboratory in each hospital. Once diagnosed, women would be treated as per standard guidelines. SGA fetus (was defined as birthweight of either twin below the 10th percentile at the same gestational age. PTB was regarded as birth before 34 + 0 weeks for twin pregnancies [16, 17]. When calculating the amount of bleeding, intraoperative blood loss was recorded by the container of suction apparatus, while postoperative bleeding volume was counted by weighing. Cesarean postpartum hemorrhage was defined as postoperative bleeding volume > 1000 ml in 24 h. These diagnostic criteria were unified in two centers before the study was initiated.

### Statistical analysis

The study was initially designed to detect 8% absolute difference in PE rate with 80% power and 5% type I error rate [18]. We assumed the incidence of PE as 4% in the LDA group, while being 12%in the control group. Accounting for a potentially 20% loss to follow-up, sample size of 424 was needed (212 in each group). A total of 1070 women met our criteria and were registered in the Cohort Management System. Actual loss to follow-up occurred in 138 women (12.9%), involving 47 women with twin-to-twin transfusion syndrome (TTTs), and the number of loss to follow-ups was less than the 20% expected, therefore we finally followed up 932 women (277 in the LDA group and 655 in the control group).

To adjust the unmatched baseline characteristics, a 1:1 propensity score matching (PSM) was applied between LDA and control groups to simulate a randomized controlled trial (RCT) of the variables. The matching algorithm was the nearest-neighbor matching and the caliper was set at 0.1. Standardized mean difference (SMD) was calculated by using R (version 2.15.3, R Foundation for Statistical Computing, Tsinghua University, China; downloaded at http://www.R-project.org/). Value of SMD ≤ 0.1 was considered to be negligible. We also performed a sensitivity analysis to evaluate biases caused by propensity score matching. SPSS version 22.0 software (SPSS Inc., Chicago, USA) was used for all statistical analyses.

We dealt with data via an independent t-test and described as mean ± standard deviation if variables were in accordance with a normal distribution. Otherwise, variables were described as median ± quartile and examined by the Kruskal–Wallis test. Differences in the classified variables were presented by percentage and evaluated by the McNemar’s test. *P* < 0.05 or SMD>0.1 were considered statistically significant. A conditional logistic regression model was used in paired data (after PSM), while an unconditional logistic model was applied in the sensitivity analysis. Confounding factors included age, Body Mass Index (BMI), and those which were previously reported to be risk factors of preeclampsia.

## Results

### Baseline characteristics

**Fig. 1:**
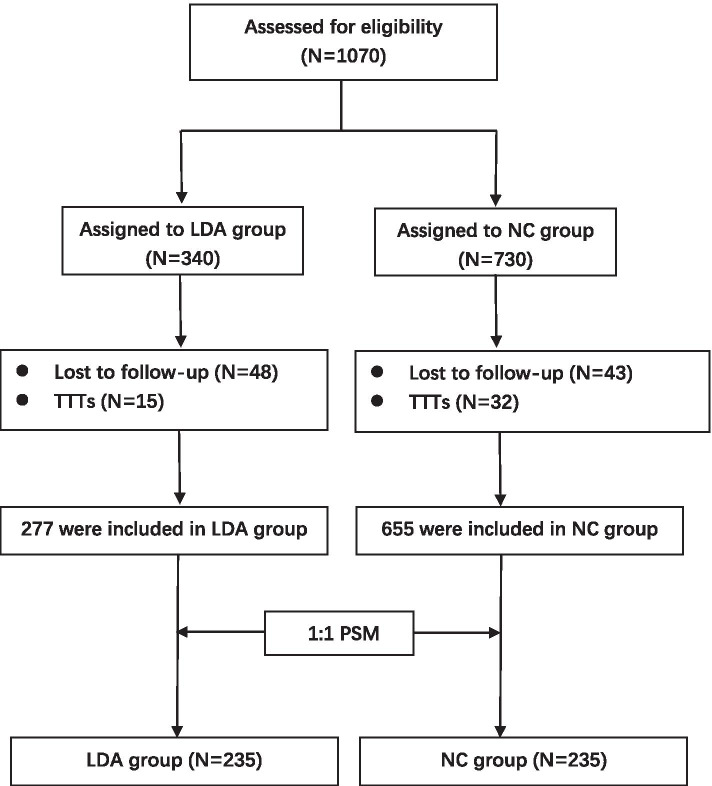
Enrollment and follow-up flow diagram

In total, 1070 twin pregnancies were recruited in this study, 138 were excluded for final analysis. Among them, 91 subjects were lost to follow-up (including 8 subjects who took extra drugs on their initiative) and 47 were diagnosed with TTTs and underwent a medical intervention. Finally, we obtained complete information from 932 pregnancies (277 in LDA, 655 in control group). (Fig. 1) Before PSM, baseline characteristics varied widely. Age in the LDA group (29.55 ± 3.93y) was younger than in the control group (30.22 ± 4.37y, *p* = 0.02) (Table 1). In the LDA group, spontaneous conception occurred in 181 cases (65.3%), which was higher than the control group (*n* = 242; 36.9%, *p* < 0.001). Moreover, the number of dichorionic twins was much higher in the NC group (86.41% vs. 56.68%, *p* < 0.001) (Table 1).

**Table 1:** Maternal and pregnancy-related characteristics before propensity score matching

Variables	LDA(***N*** = 277)	Control(***N*** = 655)	***P*** value	SMD
**Age**	29.55 ± 3.93	30.22 ± 4.37	0.022*	0.170
**Pre-Pregnant BMI**	21.45 ± 2.89	21.58 ± 2.93	0.527	0.045
**BMI Increment**	7.31 ± 2.14	7.03 ± 2.13	0.068	0.131
**Gravidity**	2 (1–3)	2 (1–3)	<0.001*	0.293
**Parity**	1 (1–1)	1 (1–1)	0.754	0.002
**Primipara**	245 (88.4)	575 (87.79)	0.826	0.021
**Methods of conception**			<0.001*	0.596
*Spontaneous*	181 (65.34)	242 (36.95)		
*IVF*	96 (34.66)	413 (63.05)		
**Chorionic type**			<0.001*	0.568
*DC*	157 (56.68)	556 (86.41)		
*MC*	120 (43.32)	99 (15.11)		
**Chronic hypertension**	2 (0.72)	6 (0.92)	1.000	0.011
**GDM**	80 (28.88)	182 (27.79)	0.750	0.024
**ICP**	27 (9.75)	83 (12.67)	0.223	0.098
**Hypothyroidism**	19 (6.86)	47 (7.18)	1.000	0.012
**Anemia**	28 (10.11)	85 (12.98)	0.230	0.035

After PSM, 235 women who received LDA treatment and 235 who did not use LDA were finally paired for analysis. Characteristics were matched well (all *p* > 0.05 & all SMD < 0.1)(Table 2).

**Table 2:** Maternal and pregnancy-related characteristics adjusted by propensity score matching

Variables	LDA(***N*** = 235)	Control(***N*** = 235)	***P*** value	SMD
**Age**	29.64 ± 3.94	29.33 ± 4.51	0.421	0.080
**Pre-Pregnant BMI**	21.50 ± 3.02	21.38 ± 3.04	0.683	0.038
**BMI Increment**	7.28 ± 2.19	7.12 ± 2.04	0.394	0.076
**Gravidity**	2 (1–3)	2 (1–3)	0.458	0.053
**Parity**	1 (1–1)	1 (1–1)	0.677	0.039
**Primipara**	207 (88.08)	204 (86.81)	0.781	0.039
**Methods of conception**				
*Spontaneous*	141 (60.0)	140 (59.57)	1.000	0.009
*IVF*	94 (40.0)	95 (40.42)		
**Chorionic type**				
*DC*	155 (65.96)	151 (64.25)	0.772	0.036
*MC*	80 (34.04)	84 (35.74)		
**Chronic hypertension**	2 (0.85)	2 (0.85)	1.000	0.000
**GDM**	65 (27.66)	63 (26.81)	0.918	0.019
**ICP**	24 (10.21)	20 (8.51)	0.635	0.056
**Hypothyroidism**	18 (7.66)	16 (6.81)	0.859	0.032
**Anemia**	25 (10.64)	29 (12.34)	0.665	0.061

### Main outcomes

In this observational cohort study (after PSM), incidence of PE was 4.7% in the LDA group vs. 9.8% in the control group and LDA showed a significant reduction of PE (RR 0.48; 95% CI 0.24–0.95). Risk of gestational hypertension was not reduced by LDA (RR 0.88; 95% CI 0.45–1.73). In subgroup analysis, LDA only reduced the risk of PE occurred before 37 weeks (RR 0.39; 95% CI 0.17–0.91) (Table 3). Median gestational length in days was larger in the LDA group than in controls (259 vs.254, *p*<0.001). Occurrence of PTB was also significantly reduced in the LDA group compared to controls (7.7% vs. 15.3%; RR: 0.50; 95% CI 0.29–0.86). Notably, LDA significantly lowered the risk of spontaneous PTB, not for iatrogenic. Protective role of LDA on SGA, however, was not strong (23.4% vs. 31.5%; RR 0.74; 95% CI 0.55–1.00). Bleeding volume after birth was 505.66 ± 300.96 ml in the LDA group and 524.57 ± 445.61 ml in controls (*p* = 0.590). I Incidence of cesarean postpartum hemorrhage was 3.4% in the LDA group and 3.8% in controls (RR 0.89; 95% CI 0.35–2.26) (Table 3).

**Table 3:** Comparisons of complications and outcomes between LDA and control groups

	LDA (***N*** = 235)	Control (***N*** = 235)	RR (95% CI)
**Hypertensive disorders**	26 (11.1)	40 (17.0)	0.65 (0.41–1.03)
Gestational hypertension	15 (6.38)	17 (7.23)	0.88 (0.45–1.73)
PE	11 (4.68)	23 (9.79)	0.48 (0.24–0.95)
*DC*	*9*	*17*	–
*MC*	*2*	*6*	–
*PE < 34 weeks*	*0*	*1*	–
*PE < 37 weeks*	*7*	*18*	0.39 (0.17–0.91)
*PE ≥ 37 weeks*	*4*	*5*	0.80 (0.22–2.94)
**Neonatal weight (mean)**	2550 (2280–2780)	2415 (2120–2650)	–
**SGA**	55 (23.40)	74 (31.49)	0.74 (0.55–1.00)
*DC*	*31*	*37*	–
*MC*	*24*	*37*	–
sSGA^a^	45/225 (20.0)	62/223 (27.80)	0.72 (0.51–1.01)
**Gestational week(d)**	259.0 (252.0–263.0)	254.0 (243.0–260.0)	–
**Preterm birth**^b^	18 (7.66)	36 (15.32)	0.50 (0.29–0.86)
Spontaneous	15 (6.38)	32 (13.62)	0.47 (0.26–0.84)
*DC*	*6*	*13*	–
*MC*	*9*	*19*	–
Iatrogenic	3 (1.28)	4 (1.70)	0.75 (0.17–3.31)
**Bleeding amount**	505.66 ± 300.96	524.57 ± 445.61	–
**Hemorrhage**	8 (3.40)	9 (3.83)	0.89 (0.35–2.26)

^a^ Excluded both of twins were growth restricted (10 cases in the LDA group, 12 cases in the Control group)

^b^ Gestational weeks less than 34

In the sensitivity analysis, the protective role of LDA on PE and PTB became undoubted, because of identical conclusions between the two methods. The effect of LDA on SGA, however, was uncertain as to the disparate outcomes before (AOR: 0.76, 95%CI: 0.45–0.91) and after PSM (Table S1, S2, S3, S4 and S5).

## Discussion

### Main findings

Early pregnancy prophylaxis with aspirin in twin pregnancies was associated with lower rates of PE and spontaneous PTB and not with an increased risk of postpartum hemorrhage. Our data appear to demonstrate that LDA prophylaxis may not be able to reduce the risk of SGA.

According to sensitivity analysis, we can conclude no obvious biases were made by PSM, and we can confirm LDA as a protector to PE and PTB. For SGA, we still considered it could also provide some benefits against SGA, and this point needs further studies to confirm. Moreover, when PSM was performed, 42 and 420 subjects were excluded in the LDA and control group. Among them, the percentage of SGA was a little more in the control group (23.8% vs. 27.4%, *p* = 0.717), and this may make some minor difference in the conclusion.

### Interpretation

Aspirin, at a daily dose as low as 60 mg, selectively and irreversibly inactivates the cyclooxygenase-1 enzyme, suppressing the production of prostaglandins and thromboxane and inhibiting inflammation and platelet aggregation in pregnancy [19]. Large meta-analyses and systematic reviews have consistently shown LDA to be effective in reducing the incidence of PE [20–24]. Benefits are generally modest and ideal candidates to receive LDA are not well-defined. Recently, the Aspirin for Evidence-based Preeclampsia Prevention (ASPRE) trial in singleton pregnancy has demonstrated that aspirin at a daily dose of 150 mg, initiated before 16 weeks of gestational age, and given at night to a high-risk population, identified by a combined first-trimester screening test, reduces the incidence of preterm pre-eclampsia by 62% compared with placebo [25]. A secondary analysis of the ASPRE trial data also demonstrated a reduction in the length of stay in the neonatal intensive care unit by 68%, mainly due to reduction in births before 32 weeks of gestational age with preeclampsia [26].

Different national guidelines have varying recommendations for LDA prophylaxis in twin pregnancy. The American College of Obstetrician & Gynecologist recommendations [27] recommends low dose aspirin (81 mg/day) initiated between 12 weeks and 28 weeks (optimally before 16 weeks) until birth, in multifetal gestations. The NICE Guidelines published in the UK [28] recommend prophylactic low dose aspirin (75 – 150 mg/day) from 12 weeks until birth of the baby if it is a first pregnancy, the mother is 40 years or older, has a pregnancy interval of 10 years, has a body mass index of > 35 kg/m^2^ or a family history of pre-eclampsia in twin or triplet pregnancies. More recently, a prospective study in twin pregnancy randomizing women to 100 mg/day aspirin versus placebo before 16 weeks noted an overall reduction in pre-eclampsia from 16 to 6% (OR 0.32; 95% CI 0.12–0.82) (a result that was increased in the cohort of twin pregnancies with high hCG levels with threshold of 29.96iu/mL) [29].

The efficacy of LDA for prophylaxis against the development of PE in singletons is proven, especially in ‘high-risk pregnancies’, but the underlying mechanisms of action are unknown. As we know, PE was mainly induced by ischemia-hypoxia of the placenta. Soluble fms-like tyrosine kinase 1 (sFlt-1) would be released by trophoblast cells when the environment was anoxic, and ischemia-hypoxia would be aggravated by high levels of sFlt-1. Recently, several studies focused on the mechanism of aspirin acting on trophoblast cells. The release of sFlt-1 could be inhibited by aspirin through diverse pathways, and the ischemia-hypoxia of the placenta would be alleviated [30–33]. It has been fully reported that the average sFlt-1 level and the sFlt-1/PlGF ratio were higher in twin pregnancies than singleton pregnancies and indicated more impairment to maternal vascular functions [34–36]. Thus, for twin pregnancies, it is more essential to protect maternal vessels. The mechanism of aspirin mentioned above provides bases for the treatment of PE and SGA, but its efficacy in multiple gestations was still uncertain because of poor clinical evidence. A meta-analysis contained 6 RCTs with 898 pregnancies has shown that using LDA can significantly reduce the risk of PE in multiple gestations, but it concluded low evidence because of limitations of each RCT [37]. Occurrence of PE in our study was lower in LDA-treated mothers and consistent with Euser et al. [29].

Whether LDA could reduce SGA has been a matter of debate [23, 24, 37]. Bergeron et al. [36] focused on multiple gestations, concluding no decreased risk of SGA by LDA (RR 1.09; 95% CI 0.80–1.47). In another meta-analysis for singletons, however, early initiated LDA could reduce the odds of SGA (RR 0.47; 95% CI 0.30–0.74) [23]. However, our data only presented a possible reduction of SGA and this conclusion still needs further studies.

Onset of PTB remains multifactorial with inflammation, uterine overdistention, or endocrine and immunological disorders [38–40]. Ischemia of placenta and vascular disorders have also been shown to contribute to the pathogenesis of PTB [41, 42]. Aspirin could down-regulate many inflammatory factors, and improve blood supply in the placenta; thus, aspirin could theoretically lower the incidence of PTB. In clinical practice, aspirin showed strong benefits in singletons preventing PTB [10, 43, 44]. In Andrikopoulou’s study, LDA was associated with a decrease only in PTB < 34w in singletons [10]. Allshouse et al. concluded LDA did not work in preventing Preterm Premature Rupture Of Membranes (PPROM) or PTB in singletons [43], although a recent RCT proved that it could, but only <34w [44]. Although the evidence in singletons was strong, there was not sufficient evidence about LDA in protecting PTB in twins. Our findings suggested that LDA could decrease the occurrence of PTB.

### Strengths and limitations

Our study has several strengths. It is an observational cohort study across two centers and has a longitudinal follow-up for each participant. As a twin pregnant cohort, we have large numbers of subjects and owned the whole information of each mother from registration to birth. We applied PSM and sensitivity analysis to deal with unmatched baseline characteristics, so conclusions were reliable. To our knowledge, this is the first cohort trial to demonstrate that early pregnancy-initiated LDA could reduce adverse outcomes in twin pregnancies.

As a cohort study based on real-world data, there were some inherent defects. No randomization was applied in this study and baseline characteristics in primary data were not matched. Although PSM was applied, we had to exclude many women for the final analysis. Secondary, it is a purely observational study, and usage of aspirin was uncontrolled, but uniformed in one medical center. Another limitation was that we did not collect other baseline information such as educational background, annual income, and so on, apart from the medical details. New multi-center RCTs should be urgently performed.

## Conclusion

Low-dose aspirin (100 mg/d) initiated at early gestational age in twin pregnancies could significantly reduce the risk of adverse outcomes, especially for preterm pre-eclampsia and preterm birth. Moreover, our data demonstrated that LDA would not increase the risk of postpartum hemorrhage.

## Supplementary Information

**Additional file 1.**

## Data Availability

The data used and analyzed during the current study are available from the corresponding author on reasonable request.

## Abbreviations

ART: Assisted Reproductive Technologies
PE: Preeclampsia
SGA: Small for gestational age
PTB: Preterm birth
PPROM: Preterm Premature Rupture Of Membranes
BMI: Body Mass Index
ACOG: American College of Obstetricians and Gynecologists
PSM: Propensity score matching
