# Supplementary material for: Low-dose aspirin for primary prevention of adverse pregnancy outcomes in twin pregnancies: an observational cohort study based on propensity score matching
Source: BMC Pregnancy Childbirth. 2021 Nov 22;21:786. doi: 10.1186/s12884-021-04217-2 (PMC8607699; doi:10.1186/s12884-021-04217-2)
Supplement: Supplementary file 1 — Additional file 1. [file 12884_2021_4217_MOESM1_ESM.docx]

**Table.S1. Univariate analysis for whole data.**

| **Variables** | **LDA(N=277)** | **Control(N=655)** | **P value** |
| --- | --- | --- | --- |
| **PE** | 13(4.69) | 71(10.84) | 0.002 |
| **Preterm birth** | 20(7.22) | 96(14.66) | 0.002 |
| **FGR** | 65(23.47) | 189(28.85) | 0.107 |
| ***sFGR*** | 54(19.49) | 162(24.73) | 0.090 |
| **Postpartum hemorrhage** | 9(3.25) | 22(3.36) | 1.000 |

**Table S2. Multivariable logistic regression analysis for PE.**

| **Variables** | **OR (95%CI)** | **Adjusted OR (95% CI) ^#^** |
| --- | --- | --- |
| **Age** | 1.06(1.01-1.12) ^*^ | 1.05(0.99-1.11) |
| **Pre-pregnant BMI** | 1.15(1.07-1.23) ^*^ | 1.17(1.08-1.26) |
| **BMI increment** | 1.22(1.10-1.35) ^*^ | 1.29(1.16-1.43) |
| **Gravidity** | 0.83(0.68-1.01) ^*^ |  |
| **Uniparas** | 3.99(1.24-12.83) ^*^ | 3.76(1.11-12.72) |
| **Spontaneous conception** | 0.40(0.24-0.66) ^*^ | 0.63(0.34-1.15) |
| **DC** | 2.42(1.23-4.77) ^*^ | 1.00(0.45-2.23) |
| **Chronic hypertension** | 5.80(2.80-11.91) ^*^ | 5.54(2.76-11.88) |
| **GDM** | 1.48(0.92-2.36) |  |
| **LDA** | 0.41(0.22-0.75) ^*^ | 0.45(0.24-0.87) |

#: Adjusted by all other characteristics marked with “*”.

**Table S3. Multivariable logistic regression analysis for PTB.**

| **Variables** | **OR (95%CI)** | **Adjusted OR (95% CI) ^#^** |
| --- | --- | --- |
| **Age** | 0.96(0.92-1.01) ^*^ | 0.97(0.92-1.02) |
| **Pre-pregnant BMI** | 1.01(0.94-1.08) |  |
| **Gravidity** | 0.97(0.84-1.13) ^*^ |  |
| **Uniparas** | 1.09(0.59-2.02) |  |
| **Spontaneous conception** | 0.94(0.63-1.39) ^*^ | 0.74(0.45-1.20) |
| **DC** | 0.67(0.44-1.04) ^*^ | 0.46(0.27-0.78) |
| **GDM** | 0.92(0.60-1.43) |  |
| **ICP** | 0.85(0.45-1.59) ^*^ | 0.88(0.46-1.68) |
| **LDA** | 0.45(0.27-0.75) ^*^ | 0.38(0.22-0.64) |

#: Adjusted by all other characteristics marked with “*”.

**Table S4. Multivariable logistic regression analysis for FGR.**

| **Variables** | **OR (95%CI)** | **Adjusted OR (95% CI) ^#^** |
| --- | --- | --- |
| **Age** | 0.98(0.95-1.01) ^*^ | 0.99(0.96-1.03) |
| **Pre-pregnant BMI** | 0.93(0.88-0.98) |  |
| **Gravidity** | 0.94(0.84-1.06) ^*^ | 0.95(0.83-1.07) |
| **Uniparas** | 0.93(0.60-1.44) |  |
| **Spontaneous conception** | 1.18(0.89-1.60) ^*^ | 1.04(0.73-1.49) |
| **DC** | 0.64(0.46-0.89) ^*^ | 0.57(0.38-0.84) |
| **GDM** | 1.10(0.80-1.51) |  |
| **LDA** | 0.76(0.55-1.05) ^*^ | 0.64(0.45-0.91) |

#: Adjusted by all other characteristics marked with “*”.

**Table S5. Multivariable logistic regression analysis for postpartum hemorrhage.**

| **Variables** | **OR (95%CI)** | **Adjusted OR (95% CI) ^#^** |
| --- | --- | --- |
| **Age** | 1.05(0.96-1.14) ^*^ | 1.02(0.93-1.13) |
| **Pre-pregnant BMI** | 1.02(0.91-1.15) |  |
| **Gravidity** | 0.95(0.71-1.26) ^*^ | 0.98(0.72-1.34) |
| **Uniparas** | 2.02(0.48-8.57) |  |
| **Spontaneous conception** | 0.48(0.22-1.06) ^*^ | 0.54(0.21-1.44) |
| **DC** | 1.62(0.61-4.27) ^*^ | 1.03(0.31-3.32) |
| **GDM** | 1.89(0.91-3.91) |  |
| **PE** | 3.78(1.63-8.73) ^*^ | 3.42(1.45-8.08) |
| **LDA** | 0.97(0.44-2.13) ^*^ | 1.34(0.57-3.13) |

#: Adjusted by all other characteristics marked with “*”.
